# Artificial neural network models to predict nodal status in clinically node-negative breast cancer

**DOI:** 10.1186/s12885-019-5827-6

**Published:** 2019-06-21

**Authors:** Looket Dihge, Mattias Ohlsson, Patrik Edén, Pär-Ola Bendahl, Lisa Rydén

**Affiliations:** 1 Department of Clinical Sciences Lund, Division of Surgery, Lund University, Lund, Sweden; 2 Department of Plastic and Reconstructive Surgery, Skåne University Hospital, Malmö, Sweden; 3 Department of Astronomy and Theoretical Physics, Division of Computational Biology and Biological Physics, Lund University, Lund, Sweden; 4 Department of Clinical Sciences Lund, Division of Oncology and Pathology, Lund University, Lund, Sweden; 5 Department of Surgery, Skåne University Hospital, SE-221 85 Lund, Sweden

**Keywords:** Breast cancer, Sentinel lymph node biopsy, Nodal status, Artificial neural networks, Prediction models

## Abstract

**Background:**

Sentinel lymph node biopsy (SLNB) is standard staging procedure for nodal status in breast cancer, but lacks therapeutic benefit for patients with benign sentinel nodes. For patients with positive sentinel nodes, individualized surgical strategies are applied depending on the extent of nodal involvement. Preoperative prediction of nodal status is thus important for individualizing axillary surgery avoiding unnecessary surgery. We aimed to predict nodal status in clinically node-negative breast cancer and identify candidates for SLNB omission by including patient-related and pathological characteristics into artificial neural network (ANN) models.

**Methods:**

Patients with primary breast cancer were consecutively included between January 1, 2009 and December 31, 2012 in a prospectively maintained pathology database. Clinical- and radiological data were extracted from patient’s files and only clinically node-negative patients constituted the final study cohort. ANN-based models for nodal prediction were constructed including 15 risk variables for nodal status. Area under the receiver operating characteristic curve (AUC) and Hosmer-Lemeshow goodness-of-fit test (HL) were used to assess performance and calibration of three predictive ANN-based models for no lymph node metastasis (N0), metastases in 1–3 lymph nodes (N1) and metastases in ≥ 4 lymph nodes (N2). Linear regression models for nodal prediction were calculated for comparison.

**Results:**

Eight hundred patients (N0, *n* = 514; N1, *n* = 232; N2, *n* = 54) were included. Internally validated AUCs for N0 versus N+ was 0.740 (95% CI = 0.723–0.758); median HL was 9.869 (*P* = 0.274), for N1 versus N0, 0.705 (95% CI = 0.686–0.724; median HL: 7.421; *P* = 0.492) and for N2 versus N0 and N1, 0.747 (95% CI = 0.728–0.765; median HL: 9.220; *P* = 0.324). Tumor size and vascular invasion were top-ranked predictors of all three end-points, followed by estrogen receptor status and lobular cancer for prediction of N2. For each end-point, ANN models showed better discriminatory performance than multivariable logistic regression models. Accepting a false negative rate (FNR) of 10% for predicting N0 by the ANN model, SLNB could have been abstained in 27.25% of patients with clinically node-negative axilla.

**Conclusions:**

In this retrospective study, ANN showed promising result as decision-supporting tools for estimating nodal disease. If prospectively validated, patients least likely to have nodal metastasis could be spared SLNB using predictive models.

**Trial registration:**

Registered in the ISRCTN registry with study ID ISRCTN14341750.

Date of registration 23/11/2018. Retrospectively registered.

**Electronic supplementary material:**

The online version of this article (10.1186/s12885-019-5827-6) contains supplementary material, which is available to authorized users.

## Background

Sentinel lymph node biopsy (SLNB) is the standard axillary staging procedure for patients with clinically node-negative primary breast cancer. In the majority of patients, SLNB will prove negative and no nodal metastasis is diagnosed [1]. Moreover, in approximately half of the SLNB-positive cases, no further metastatic lymph nodes will be harvested during routine completion axillary lymph node dissection (ALND) [2]. While a prospective randomized trial [3] questioned the value of axillary surgical staging in selected low-risk patients, the American College of Surgeons Oncology Group (ACOSOG) Z0011 trial suggested that patients with 1–2 sentinel node metastasis were eligible for minimalistic axillary surgical interventions without completion ALND, and reported no negative consequences for survival or locoregional recurrence after 10 years of follow-up [4, 5]. However, patients with heavy-burden axillary disease (stage N2) could benefit from preoperative selection for neoadjuvant therapy or direct ALND. An improvement in breast cancer management has been to seek an individualized surgical approach to the axilla, and an accurate prediction of the axillary status preoperatively would facilitate individualized surgical decisions.

However, validation of prediction models for nodal status have shown diverse accuracies in estimating nodal involvement [6, 7] and mirror the complexity of factors related to axillary metastasis, and the paucity of models to analyze nonlinear dynamics between relevant variables. Artificial neural networks (ANNs) are nonlinear machine learning methods proposed as supplements to standard statistical models for predicting multifaceted biological events [8, 9], and help in the exploration of underlying nonlinear interactions of interconnected predictors [10]. ANNs have gained utility in various clinical settings, and are being used as diagnostic and prognostic tools in cancer [11, 12], and for prediction of surgical outcomes in various disease conditions [13, 14].

The primary aim of this study was to utilize commonly available patient-related and clinicopathological characteristics in ANN modeling to predict nodal axillary status. The end-points were chosen to reflect the extent of nodal metastatic burden, with an aim to designate no lymph node metastasis (N0), metastases in 1–3 lymph nodes (N1) and metastases in ≥ 4 lymph nodes (N2), respectively. A secondary aim was to assess possible clinical benefit in detecting disease-free axilla (N0). Patient stratification preoperatively using the ANN model applying nodal predictive variables would help identify patients least likely to benefit from SLNB, consequently reducing the rate of unbeneficial surgery. In the clinical setting, the models may be useful tools for risk-benefit analysis of axillary treatment and contribute to improved patient stratification for surgical axillary interventions.

## Methods

### Patient selection

**Fig. 1:**
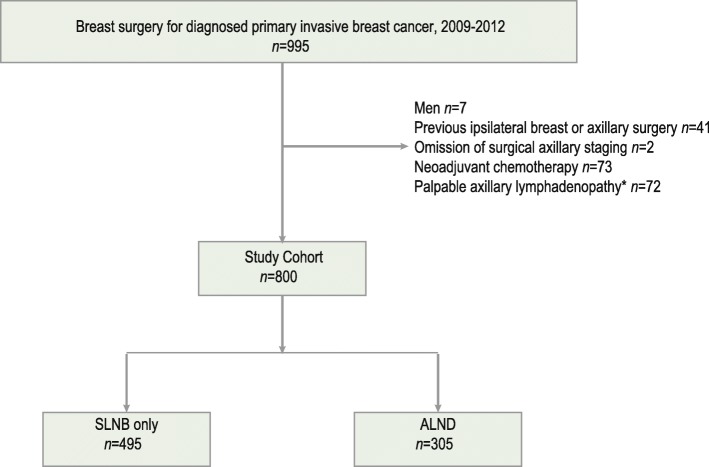
Study population. The flow chart shows the original patient population, excluded patients, and details of the surgical axillary nodal staging procedures. *Abbreviations: SLNB*, Sentinel lymph node biopsy; *ALND*, Axillary lymph node dissection. * Palpable adenopathy or matted lymph nodes at the time of diagnosis

Patients (*n* = 995) were included in a prospectively maintained pathological database and the following eligibility criteria were applied: consecutive patients diagnosed with primary breast cancer between January 2009 and December 2012 at the Skåne University Hospital (Lund, Sweden). Exclusion criteria were: male sex, previous ipsilateral breast or axillary surgery, previous neoadjuvant therapy, palpable axillary lymphadenopathy (palpable adenopathy or matted lymph nodes at the time of diagnoses) and omission of standard axillary staging procedure by SLNB or ALND (Fig. 1). Presence of micro (> 0.2 mm and/or more than 200 cells, but none > 2.0 mm)- or macrometastases (> 2.0 mm) on SLNB indicated axillary node-positivity. Patients gave verbal informed consent to participate at time of diagnosis and the ethics committee at Lund University approved this procedure (LU 2013/340). Patients were informed that they had the opportunity to opt-out if they were not willing to participate in the study.

### Data collection

Data regarding previous breast or axillary surgery and mode of detection (mammography screening or symptomatic presentation) were obtained from The Swedish National Quality Registry for Breast Cancer and from the public mammography screening program records. Medical records were reviewed for age, menopausal status, clinical axillary status, and body mass index (BMI) data. A breast pathologist extracted the following histopathological variables: synchronous bilateral malignancy status, tumor localization in the breast (centrally or in quadrants, overlapping lesions were allocated equally into adjacent quadrants for analysis), multifocality (two or more tumor foci separated by benign breast tissue; multicentricity was not a separate entity), tumor size, histological type (ductal carcinoma of no special type, invasive lobular carcinoma, or other invasive carcinoma), histological grade, biomarker status (estrogen receptor (ER), progesterone receptor (PR), and human epidermal growth factor receptor 2 (HER2)), Ki-67 positivity, and lymphovascular invasion (LVI) status.

### Acquisition of the ANN classifiers

Three ANN models were defined, each containing multi-layer perceptrons (MLPs) with three layers: an input layer corresponding to the number of risk variables (patient-related and clincopathological), one hidden layer, and a single node output layer. The chosen output reflected the extent of metastatic involvement: disease-free axilla (N0), N1 (1–3 metastatic nodes versus N0), and N2 (*N* ≥ 4 metastatic nodes versus N0 and N1).

**Fig. 2:**
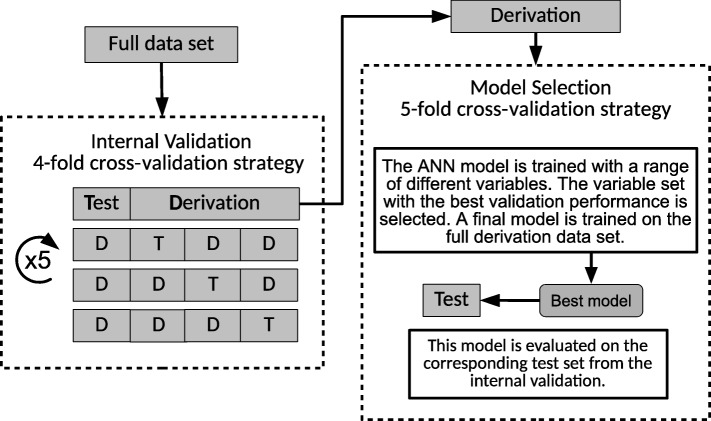
Model selection and internal validation strategies. Internal validation was performed by 4-fold cross-validation, which was repeated five times. Each round of cross-validation involved partitioning the data into a test set and a derivation set. The model selection was carried out for each of the derivation sets, independent of the corresponding test set, and was aimed to minimize information leakage. Model selection strategy was based on a 5-fold cross-validation, which was repeated seven times. *Abbreviations: D,* Different parts of the derivation set in each round of cross-validation. *T*, The test set in each round of cross-validation

An ensemble technique was applied; several ANNs were averaged into a single prediction model for each classification output. Each individual ANN in the ensemble was trained by standard back-propagation techniques using a cross-entropy error function [8] to learn the association to a given nodal status output. To avoid overfitting, the dropout technique [15, 16] was employed on the input layer. Internal model validation strategy was performed by 4-fold cross-validation, which was repeated five times. Missing data were handled by multiple random imputations for each of the five repetitions. This model validation strategy generated (5 × 4) 20 derivation sets and 20 test sets. For each of the derivation sets, a model selection procedure was carried out independently of the corresponding test set. The model selection strategy was based on a 5-fold cross-validation, which was repeated seven times. Model selection identified the best set of variables (dropout probability and number of hidden nodes) using a grid search. The model validation and selection procedures (Fig. 2) minimized information leakage as each test set did not influence the model selection in any way.

In total, 20 ANN ensemble models were trained and evaluated for each of the nodal status outputs. An ANN ensemble consisted of 15 individually trained MLPs, and the average of these networks was used as the ensemble output. Each of the MLPs was allowed to vary in size during model selection.

### Predictive performance and statistical analysis

The performance of the ANN-based models to classify nodal status outputs was assessed by area under the receiver operating characteristic curve (AUC). Discriminatory performances were compared with multivariable logistic regression, using identical model test sets and risk variables, where differences were evaluated by comparing mean validation AUC values (Wilcoxon signed-rank test). Logistic regression analysis was performed and odds ratios (mean odds ratios calculated as per Lippman et al) [17], were used to quantify the association of a risk variable with the outcome. The overall importance of selected risk variables in each classification model was assessed by means of a permutation technique [18]. In short, a predictor variable was randomized across the evaluation cohort and the effect of this randomization on the estimated performance was measured. The predictor associated with the largest decrease in performance was assigned an importance value of 1. All other variables were assigned a position in this list based on the associated decrease in performance upon randomization.

Negative predictive value (NPV) and false negative rate (FNR) are two separate but related principles to assess the usefulness of a predictive tool. While the NPV indicates the probability that a patient with predicted disease-free axilla will be truly free of axillary disease, the FNR depicts the deficiency of the model in predicting nodal spread as a ratio relative to all pathology-defined nodal metastases [19]. A cut-off for classification of nodal-negativity was set based on maximized NPV in the ANN model for N0. As previously described [18], this threshold was aimed at identifying individuals with a very low probability of axillary disease. True positive (TP), true negative (TN), false positive (FP), and false negative (FN) results were assessed to evaluate the following: SLNB reduction rate = (TN + FN)/(TN + FN + TP + FP), and FNR = FN/FN + TP. Alternative cut-offs at FNR 5 and 10% were also applied and corresponding possible SLNB reduction rates were calculated.

The distribution of clinicopathological characteristics across the nodal classification outputs were evaluated by the Jonckheere-Terpstra test, Chi Square test for trend, Pearson Chi Square test, or the Fisher’s exact test, as appropriate. Hosmer-Lemeshow goodness-of-fit (HL) statistic was used to assess calibration. All analyses were performed with IBM SPSS Statistics for Windows (version 24.0) and with custom-made software written in C (gcc version 4.8.5), and Perl (version 5.18.2).

The developing of the predictive models and the reporting of the findings were in accordance with an EQUATOR Guideline for reporting machine learning predictive models [20], which supported the STROBE statement for the reporting of observational studies [21].

## Results

### Study population

Figure 1 displays the axillary surgical procedures performed for the overall study cohort (*n* = 800). The nodal status distribution was as follows: N0: 514 (64%); N1: 232 (29%); and N2: 54 (7%). Clinical and histopathological characteristics are summarized in Table 1.

**Table 1:** Patient and tumor characteristics

	All (*n* = 800)	N0 (*n* = 514)	N1 (*n* = 232)	N2 (*n* = 54)	*p value*
Age^a^, years	64 (24–92)	64 (33–92)	63 (24–92)	62 (26–85)	0.039^b^
Missing	0	0	0	0	
BMI^a^, kg/m^2^	25 (16–49)	26 (16–44)	26 (16–46)	26 (20–49)	0.569^b^
Missing	41	24	13	4	
Menopausal status					0.011^c^
Premenopausal	130 (17)	70 (14)	48 (22)	12 (24)	
Postmenopausal	628 (83)	414 (86)	175 (78)	39 (76)	
Missing	42	30	9	3	
Mode of detection					< 0.001^c^
Mammographic screening	457 (57)	321 (63)	113 (49)	23 (43)	
Symptomatic presentation	343 (43)	193 (38)	119 (51)	31 (57)	
Missing	0	0	0	0	
Bilateral cancer					0.255^c^
Absent	770 (96)	499 (97)	222 (96)	49 (91)	
Present	30 (4)	15 (3)	10 (4)	5 (9)	
Missing	0	0	0	0	
Multifocality					< 0.001^c^
Absent	610 (77)	418 (82)	161 (71)	31 (61)	
Present	179 (23)	92 (18)	67 (29)	20 (39)	
Missing	11	4	4	3	
Tumor site, quadrants of breast					0.170^d^
Central C501	22 (3)	14 (3)	6 (3)	2 (4)	
Upper inner C502	108 (14)	78 (15)	25 (11)	5 (9)	
Lower inner C503	46 (6)	32 (6)	12 (5)	2 (4)	
Upper outer C504	266 (33)	165 (32)	86 (37)	15 (28)	
Lower outer C505	84 (11)	43 (8)	31 (13)	10 (19)	
Overlapping lesions: 3, 6, 9, 12 o’clock	274 (34)	182 (35)	72 (31)	20 (37)	
Missing	0	0	0	0	
Tumor size^a^, mm	15 (0.5–90)	13 (0.5–70)	17 (0.9–90)	23 (6–70)	< 0.001^b^
Missing	1	0	1	0	
Histological type	800	514	232	54	0.010 ^e^
Ductal	640 (80)	408 (79)	195 (84)	37 (69)	
Lobular	101 (13)	60 (12)	27 (12)	14 (26)	
Other	59 (7)	46 (9)	10 (4)	3 (6)	
Missing	0	0	0	0	
Histological grade					0.003^c^
I	197 (25)	142 (28)	48 (21)	7 (13)	
II	366 (46)	231 (46)	106 (46)	29 (54)	
III	229 (29)	134 (26)	77 (33)	18 (33)	
Missing	8	7	1	0	
Vascular invasion					< 0.001^c^
Absent	545 (85)	401 (94)	124 (72)	20 (37)	
Present	94 (15)	28 (7)	48 (28)	18 (33)	
Missing	161	85	60	16	
ER status					0.020 ^c^
Positive	729 (91)	459 (90)	218 (94)	52 (96)	
Negative	69 (9)	53 (10)	14 (6)	2 (4)	
Missing	2	2	0	0	
PR status					0.075^c^
Positive	673 (84)	421 (82)	206 (89)	46 (85)	
Negative	125 (16)	91 (18)	26 (11)	8 (15)	
Missing	2	2	0	0	
HER2 status					0.208^c^
Positive	86 (12)	51 (11)	27 (13)	15 (16)	
Negative	647 (88)	422 (89)	184 (87)	41 (84)	
Missing	67	41	21	5	
Ki-67 ^a^, %	15 (0–94)	14 (0–94)	17 (1–81)	20 (3–76)	0.001^b^
Missing	48	29	16	3	

*p* values refer to the comparisons between N0, N1 and N2

Column percentages are given for categorical variables unless indicated otherwise

Percentage have been rounded and may therefore not total 100

*Abbreviations: N0* Lymph node-negative, *N1* Lymph node metastasis involving 1–3 nodes, *N2* Lymph node metastasis involving at least 4 nodes, *BMI* Body mass index, *ER* Estrogen receptor, *PR* Progesterone receptor*, HER2* Human epidermal growth factor receptor

^**a**^median (range)

^b^Jonckheere-Terpstra Test

^c^χ2 test for trend

^d^Pearson χ2 test

^e^Fisher’s exact test

### Predictive Clinicopathological variables

**Fig. 3:**
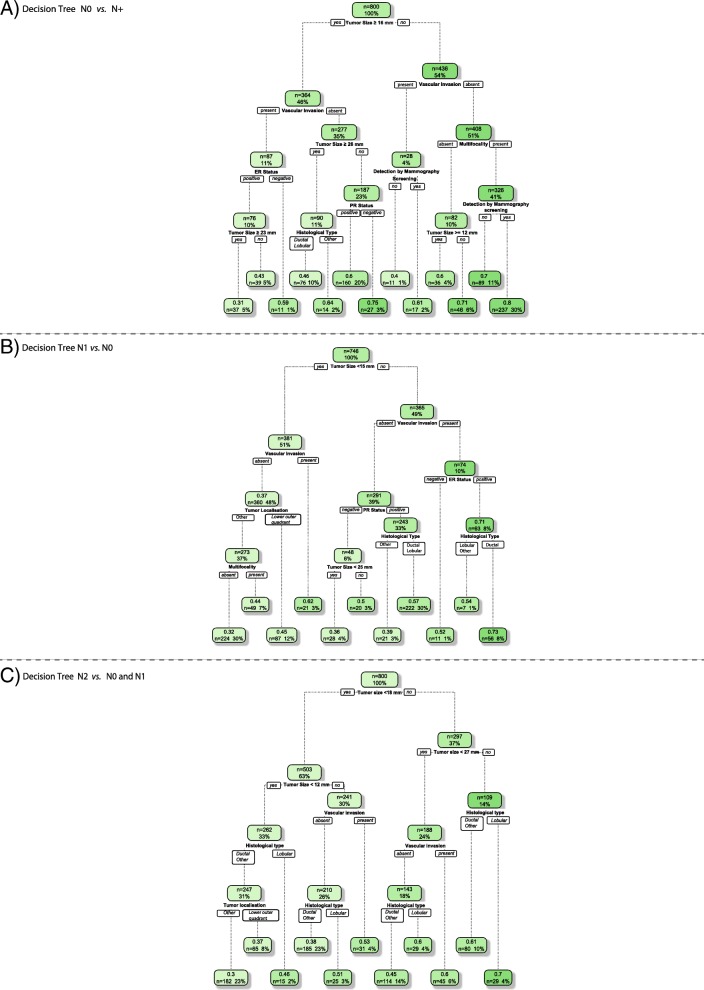
Simplified illustration of the decision made by the ANN model. The regression trees were trained to predict the output probabilities made by the ANN model, given the identified top-ranked variables. Each tree was only allowed to grow to depth 4 and the full dataset was used to construct the trees. The numbers in the green boxes indicate average ANN output and the size of the data at the given node. **A** Decision tree N0 vs. N+; **B** Decision tree N1 vs. N0; **C** Decision tree N2 vs. N0 and N1

Table 1 displays the 15 potential clinicopathological risk variables for designation of nodal status (as N0, N1, or N2). Although the discriminatory effect of each variable cannot be expressed in terms of straightforward coefficients, mean odds ratios and sensitivity analysis can facilitate the interpretation of the relationship between an independent variable and the output. Table 2 displays selected variables, ranks, and mean odds ratios used for classification of each nodal status output. The ANN structure for predicting disease-free axilla status was characterized by a complex integration of predictors as input variables. The top ten variables were tumor size, LVI, multifocality, ER status, histological type, PR status, mode of detection, age, tumor localization in the breast, and Ki-67 positivity. To discriminate low-burden disease (N1), the same top variables were selected, with two exceptions: omission of mode of detection, and inclusion of menopausal status. While tumor size and LVI remained the top two variables most strongly associated with any nodal status output, other variables varied in rank of association with N0, N1, and N2 disease. Only six input variables were found to be predictive in the ANN structure for heavy-burden disease (N2): tumor size, LVI, ER status, histological type, and multifocality. A simplified illustration of the importance and relations of the different input variables as regression trees was constructed, for each of the models, and depicted in Fig. 3. Each tree was trained to predict the output (probabilities) of the corresponding ANN model. Sensitivity analysis of the assigned importance of the top rank predictive variables linearly scaled into a summation of 1 for the three models are given in Additional file 1.

**Table 2:** Top rank predictive clinicopathological variables in the ANN models for each of the axillary nodal status outcome

Predictors	Rank^a^	OR^b^
N0 vs. N+ (*n* = 800)		
Tumor size, per mm	100.00	0.950 (0.917–0.984)
Vascular invasion, present vs. absent	40.94	0.409 (0.201–0.681)
Multifocality, present vs. absent	14.61	0.670 (0.452–0.910)
ER status, positive vs. negative	10.49	0.618 (0.312–1.110)
Histological type	9.98	
Ductal		1 [reference]
Lobular		1.092 (0.692–1.688)
Other		2.033 (1.112–3.751)
PR status, positive vs. negative	9.60	0.678 (0.443–0.962)
Mode of detection, mammographic screening vs. symptomatic presentation	7.97	1.310 (0.987–1.705)
Age, per year	6.76	1.010 (0.997–1.024)
Tumor localization in the breast^c^	6.47	
Upper outer quadrant		1 [reference]
Central		1.137 (0.592–2.099)
Upper inner quadrant		1.323 (0.922–1.889)
Lower inner quadrant		1.112 (0.500–2.034)
Lower outer quadrant		0.680 (0.383–1.039)
Ki67, percentage	5.07	0.996 (0.981–1.009)
N1 vs. N0 (*n* = 746)		
Tumor size, per mm	100.0	1.050 (1.016–1.087)
Vascular invasion, present vs. absent	46.15	2.492 (1.440–4.376)
Multifocality, present vs. absent	16.47	1.527 (1.101–2.180)
PR status, positive vs. negative	14.36	1.613 (1.058–2.409)
Histological type	10.55	
Ductal		1 [reference]
Lobular		0.785 (0.466–1.131)
Other		0.491 (0.242–0.872)
ER status, positive vs. negative	9.04	1.657 (0.813–2.895)
Age, per year	6.95	0.992 (0.978–1.003)
Tumor localization in the breast^c^	6.03	
Upper outer quadrant		1 [reference]
Central		0.805 (0.324–1.415)
Upper inner quadrant		0.741 (0.510–1.080)
Lower inner quadrant		0.974 (0.476–1.759)
Lower outer quadrant		1.462 (0.915–2.369)
Ki67, percentage	5.07	1.002 (0.992–1.014)
Menopausal status, postmenopause vs. premenopause	4.88	0.783 (0.504–1.065)
N2 vs. N0 and N1 (*n* = 800)		
Tumor size, per mm	100.0	1.039 (1.020–1.054)
Vascular invasion, present vs. absent	36.71	1.805 (1.345–2.451)
ER status, positive vs. negative	13.25	1.777 (1.357–2.873)
Histological type	4.55	
Ductal		1 [reference]
Lobular		1.658 (1.252–2.083)
Other		0.910 (0.401–1.194)
Tumor localization in the breast^c^	3.48	
Upper outer quadrant		1 [reference]
Central		1.208 (0.739–2.033)
Upper inner quadrant		1.012 (0.778–1.270)
Lower inner quadrant		1.077 (0.684–1.410)
Lower outer quadrant		1.544 (1.134–2.021)
Multifocality, present vs. absent	2.32	1.219 (1.033–1.338)

*Abbreviations: N0* Lymph node negative, *N+* Any lymph node metastasis, *N1* Lymph node metastasis involving 1–3 nodes, *N2* Lymph node metastasis involving at least 4 nodes, *ER* Estrogen receptor, *PR* Progesterone receptor

^a^Sensitivity analysis, linearly scaled into percentage

^b^Mean odds ratio, values enclosed by parentheses represent 90% central range defined by the 5th and 95th percentiles

^c^Tumor localization in the breast was classified in quadrants or defined as centrally located; overlapping lesions were equally allocated into adjacent quadrants

### Discriminatory ability and calibration

Mean training AUC from the derivation sets was 0.735 for disease-free axilla. The corresponding internally validated AUC for N0 was 0.740 (95% confidence interval (CI) = 0.723–0.758) with an observed median HL statistic of 9.869 (*P* = 0.274). The ANN model to distinguish low-burden disease (N1 versus N0) showed an AUC of 0.706 in the training set, and an internally validated AUC of 0.705 (95% CI = 0.686–0.724). For high-burden disease (N2 versus N0 and N1), the training AUC was 0.735, while the internally validated AUC was 0.747 (95% CI = 0.728–0.765), with the corresponding median HL statistic values of 7.421 (*P* = 0.492) and 9.220 (*P* = 0.324), respectively. This indicated that the number of N0, N1, and N2 cases observed were not significantly different from those predicted by the models, and that the overall model calibration was good.

### Performances in comparison to linear multivariable logistic regression

The discriminative abilities of internally validated ANN models and cross-validated multivariable logistic regression models (MLR) were compared. To distinguish N0, MLR models achieved a mean AUC of 0.727 (95% CI = 0.708–0.746). In 17 out of 20 test sets for N0, the AUC values from ANN models were greater than those obtained from the corresponding MLR models (*P* < 0.001). For N2 classification, MLR models obtained a mean AUC of 0.723 (95% CI = 0.694–0.750). Here, AUC values from the ANN models were greater than those obtained from the matching MLR models in 16 out of 20 test sets (*P* = 0.003). Likewise, the ANN models for N1 classification achieved greater discriminatory ability than did the corresponding MLR models in the majority (14 out of 20) of test sets (*P* = 0.040). However, an equivalent mean AUC of 0.700 (95% CI = 0.678–0.720) was obtained from the MLR models. Comparing ANN and MLR models based on the HL statistics, the ANN models were significantly more calibrated for N1 and N2 models (*P* = 0.003 and *P* = 0.006, respectively). However, for the N0 model the difference, in favor of the ANN model, was not significant (*P* = 0.09).

### Clinical utility for SLNB reduction

To assess the clinical utility of ANN models in reducing unnecessary SLNB procedures, prediction of N0 status using a NPV-oriented cut-off was assessed. The maximized NPV was 95%. If the N0 model with this threshold were to be used in a preoperative setting to identify N0 patients, the ANN model would reduce SLNB procedures by 7.50%, with a corresponding FNR of 1.05%. If an alternative FNR of 5–10% was to be accepted, in accordance with the FNR for sentinel node biopsy technique, the corresponding SLNB reduction rate would be 17.75–27.25% (Table 3).

**Table 3:** SLNB reduction rates using the ANN model to predict disease-free axilla. Possible SLNB reduction rate corresponding to cut-offs at maximum negative predictive value, false negative rate 5 and 10%, respectively

	N0 vs. N+ n = 800			
Cut-off Max NPV 0.95	TP	TN	FP	FN
No.	283	57	457	3
SLNB Reduction Rate	(TN + FN) / (TP + TN + FP + FN) = 7.50%			
False Negative Rate	FN / (TP + FN) = 1.05%			
Cut-off NPV 0.90	TP	TN	FP	FN
No.	272	128	386	14
SLNB Reduction Rate	(TN + FN) / (TP + TN + FP + FN) = 17.75%			
False Negative Rate	FN / (TP + FN) = 5%			
Cut-off NPV 0.87	TP	TN	FP	FN
No.	258	190	324	28
SLNB Reduction Rate	(TN + FN) / (TP + TN + FP + FN) = 27.25%			
False Negative Rate	FN / (TP + FN) = 10%			

*Abbreviations: N0* Lymph node negative, *N+* Any lymph node metastasis, *SLNB* Sentinel lymph node biopsy, *Max NPV* Maximum negative predictive value, *TP* True positive*, TN* True negative*, FP* False positive*, FN* False negative

## Discussion

The current study presents ANN-based models for the prediction of nodal status based on routinely available clinicopathological characteristics, in a cohort of primary breast cancer patients consecutively and prospectively included in a pathology database. Internally validated performances displayed AUCs ranging from 0.705–0.747, with good calibration. These models highlighted the utility of nonlinear assessments of clinical characteristics and histological variables for prediction of axillary nodal status, especially in distinguishing disease-free axilla (N0) from high-burden disease (N2).

ANN models have been useful in detecting nodal metastasis on histopathological slides [22], and in evaluating risk of non-sentinel node involvement in breast malignancies [23]. Previous studies have proposed ANN-based algorithms for predicting nodal metastasis [24–26], though these studies were either based on small sample sizes or were conducted in selected patient cohorts. To the best of our knowledge, this is the largest study to present ANN-based algorithms predicting the extent of nodal metastatic burden in a population-based, contemporary, breast cancer cohort.

To predict nodal metastasis, our model integrates a complex set of input variables which reflect the multifactorial nature of the axillary metastatic process [27]. Variables in ANN-based models should not be taken for independent since the cause and effect reflect a dynamic process. Nevertheless, an attempt was made to better comprehend the importance of each variable by sensitivity analysis. While mean odds ratios were used for simplicity, the corresponding percentiles emphasized the dynamic nature of the input variables.

The present results reinforced tumor size [28] and LVI [29] as the most significant predictors of axillary metastasis. Age was significant in predicting disease-free axilla and low-burden disease. A nonlinear association between age and nodal status has previously been shown, with a low probability of nodal metastasis in those aged< 70 years, and increased probability in those aged > 70 years [30, 31]. In this study, positive ER and PR status was predictive of nodal metastasis, in agreement with literature; the TNBC subtype, although more aggressive, infrequently metastasizes to the axilla [32]. While a negative PR status has been shown to independently lower the risk of nodal metastasis [33], Ki-67 positivity has been associated with nodal metastasis [34, 35], and the present results are in agreement. Interestingly, alterations in the distribution of the breast cancer intrinsic subtypes has been reported to occur from the premenopausal state to the postmenopausal state [36, 37]. It is also noteworthy that menopausal status, in addition to hormone receptor status and age, was predictive of disease burden. Some publications have suggested a higher proportion of nodal metastasis in lobular cancer than that in the breast carcinoma of no special (ductal) type [38]; however, others have either found no significant differences [39], or have implied a lower incidence of nodal metastasis in the lobular than that in the ductal type [40]. The present results revealed a nonlinear association between histological type and nodal status. As supported by previous reports, the upper-outer quadrant localization of the tumor was the most common [41], and multiple lesions were predictive of axillary metastasis [33]. In accordance with published data [42], a tumor location in the inner quadrants, in comparison with that in the upper-outer quadrants, was predictive of disease-free axilla. However, medial tumor localization has also been related to increased risk of relapse [43]. Differences in lymphatic drainage patterns from the breast have been reported between palpable and nonpalpable lesions [44]. Of note, about 63% of the cases in the current cohort were diagnosed by mammography screening, and mode of detection was a significant factor in the prediction of axillary metastasis. Although the value of mammography screening is extensively debated [45], the current findings supported the notion that mode of detection complements information on tumor features and biology [46].

The ACOSOG Z0011 trial results [4, 5], were supportive of less extensive axillary surgery in patients with 1–2 metastatic sentinel nodes [47], and underlined the importance of distinguishing between low- and high-burden metastatic involvement; accordingly, ALND was omitted in women with cT1-2 N0 disease, and those with < 3 positive sentinel nodes underwent breast-conserving therapy with adjuvant treatments. As with the ACOSOG Z0011 trial, a negative clinical axillary status was a criterion in the present study. However, eligibility criteria for the current study were independent of the surgical intervention to the breast (mastectomy or breast-conserving surgery).

Predicting low-burden disease (≤3 metastatic nodes) was more challenging than was predicting disease-free axilla or high-burden disease. Nevertheless, identifying presence of metastatic burden is valuable. On an average, 2–3 lymph nodes are removed if SLNB alone is performed for nodal staging [48] and most metastatic nodes are identified with the excision of the first three sentinel nodes [49]. To improve the accuracy of predicting 1–3 metastatic lymph nodes, inclusion of imaging features from magnetic resonance imaging into the model might be beneficial. However, MRI is not always available in the preoperative setting whereas the chosen predictors in our model are. An accurate preoperative prediction of ≤3 metastatic nodes could provide clinicians with important information supporting SLNB staging procedure but spare a majority patients from completion ALND. For patients predicted to have N2 disease, the option of neoadjuvant therapy or upfront ALND can be discussed with the patient. The proportion of patients with node-positive disease is declining, and alternative non-invasive methods to surgical staging are increasingly being explored and our prediction model of N0 aims to add knowledge in this field. With a cut-off at maximized NPV to identify those with disease-free axilla, 7.50% of patients would be spared unnecessary SLNB. In comparison, the reported FNR for the SLNB procedure has been 5–10% [1, 48] and applying these FNR cut-offs for current N0 prediction would bring the SLNB reduction rate to 17.75–27.25%. While adopting the clinically accepted false negative rate of 10% for the SLNB procedure, nearly one third of all node negative patients with a predicted N0 by the model could have been spared a surgical staging procedure.

The present study has several limitations. Besides its retrospective nature, the models were developed from a single-center cohort. Furthermore, high-burden axillary metastasis was uncommon, which impacts the generalizability of the outcome. However, the cohort originated from a prospectively maintained database, which represents a contemporary population with access to a well-established public mammography-screening program. On the other hand, the possibility to obtain information on the risk variables from a monitored source strengthened the study. Unlike results relying on diverse registries, all histopathological characteristics analyzed in the study were managed by a single breast pathologist, which helped to minimize inaccuracies. However, the optimal preoperative utility of the models requires key variables such as LVI, which may not always be achievable on core-needle biopsy [50]. Although meticulous internal validation was performed with results supporting model robustness, further external validation in an independent cohort is necessary to confirm the utility of the models as guidance tools.

## Conclusions

The current study showed that nodal status is related to several independent patient and tumor characteristics, and that a nonlinear association exists between preoperatively obtainable clinicopathological variables and degree of axillary metastatic involvement. ANN models proved especially favorable in distinguishing high-burden disease and disease-free axilla and could thus be useful as a clinical decision tool in the preoperative setting imputing selected risk variables. If a threshold for classification of node-negativity were applied for high NPV and low FNR, individuals with a very low probability of axillary disease would not have been selected for SLNB by the model, and would be spared from unbeneficial axillary surgery.

## Additional file

Additional file 1: Sensitivity analysis of the assigned importance of the top rank predictive variables for the three models. Sensitivity analysis of the assigned importance of the top rank predictive variables for N0 vs. N+, N1 vs. N0 and N2 vs N0 and N1 linearly scaled into a summation of 1. (PDF 1083 kb)

## Data Availability

The datasets used and/or analysed during the current study are available from the corresponding author on reasonable request.

## Abbreviations

ACOSOG: American College of Surgeons Oncology Group
ALND: Axillary lymph node dissection
ANN: Artificial neural network
AUC: Area under the receiver operating characteristic curve
BMI: Body mass index
ER: Estrogen receptor
FN: False negative
FNR: False negative rate
FP: False positive
HER2: Human epidermal growth factor receptor 2
HL: Hosmer-Lemeshow goodness-of-fit test
LVI: Lymphovascular invasion
MLPs: Multi-layer perceptrons
MLR: Multivariable logistic regression models
NPV: Negative predictive value
PR: Progesterone receptor
SLNB: Sentinel lymph node biopsy
TN: True negative
TP: True positive
