# Supplementary figures and images for: Artificial neural network models to predict nodal status in clinically node-negative breast cancer
Source: BMC Cancer. 2019 Jun 21;19:610. doi: 10.1186/s12885-019-5827-6 (PMC6588854; doi:10.1186/s12885-019-5827-6)

**Sensitivity Analysis N0 vs.N+**

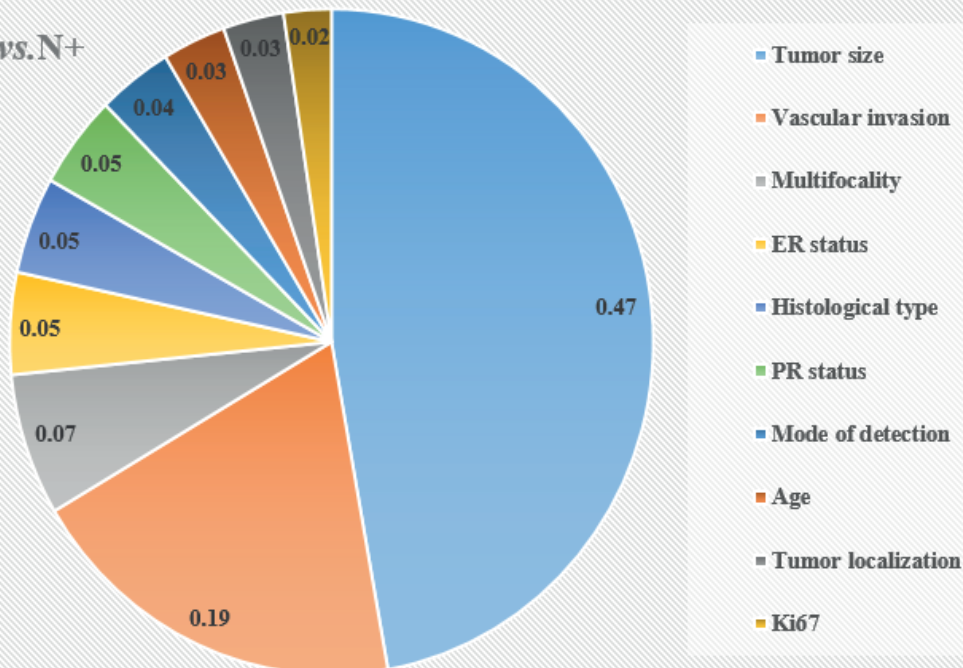

**Sensitivity Analysis N1 vs. N0**

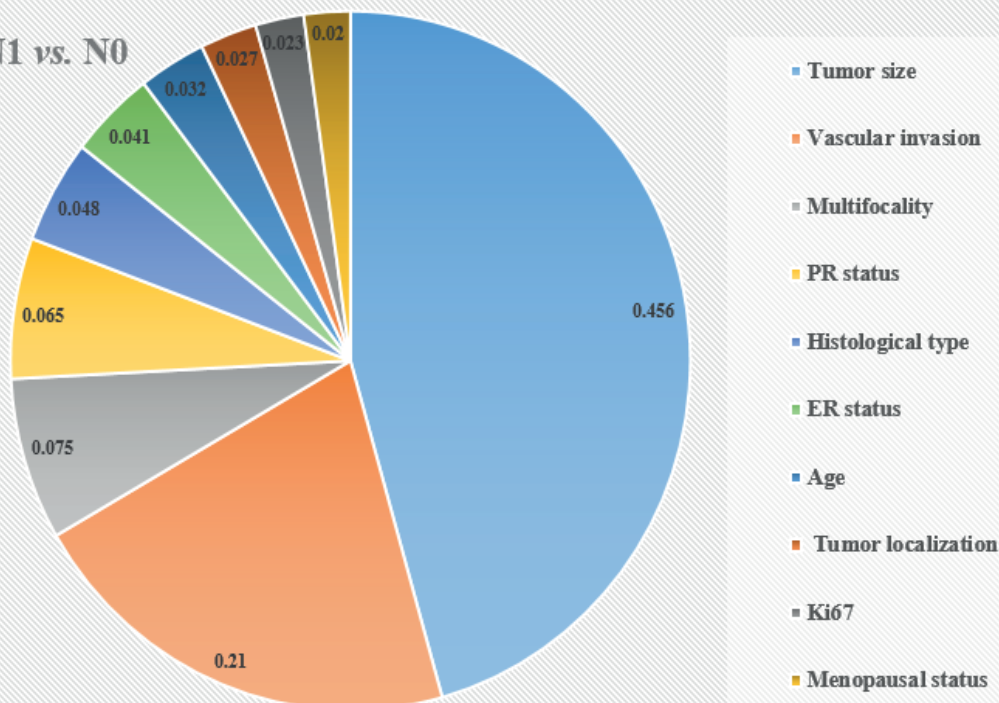

**Sensitivity Analysis N2 vs. N0 and N1**

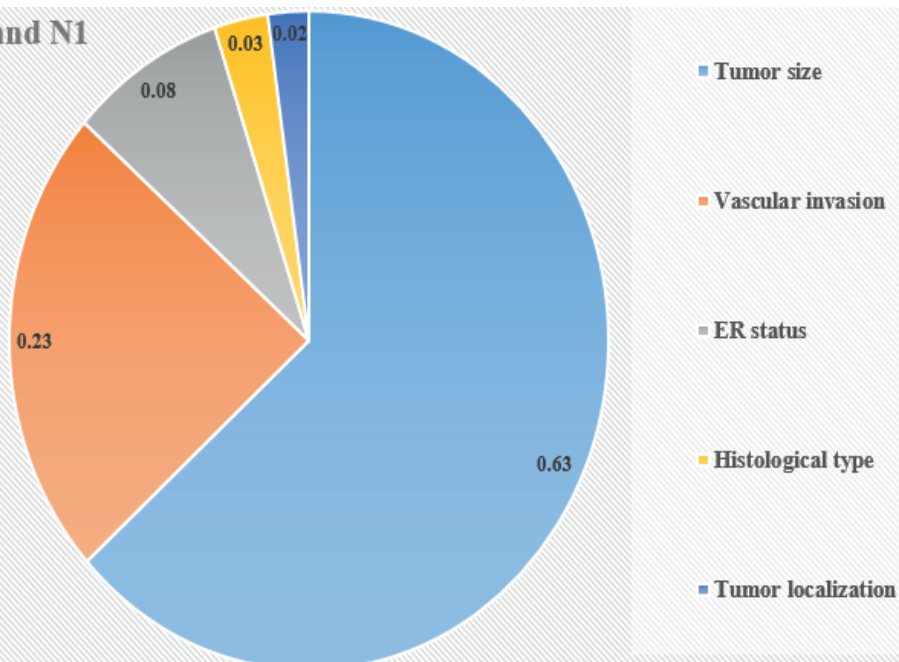

Supplement: Supplementary file 1 — Sensitivity analysis of the assigned importance of the top rank predictive variables for the three models. Sensitivity analysis of the assigned importance of the top rank predictive variables for N0 vs. N+, N1 vs. N0 and N2 vs N0 and N1 linearly scaled into a summation of 1. (PDF 1083 kb) [file 12885_2019_5827_MOESM1_ESM.pdf]
